# Natural Killer T Cell Intravascular Lymphoma With Presentation of Musculoskeletal Pain: A Case Report

**DOI:** 10.7759/cureus.20711

**Published:** 2021-12-26

**Authors:** Shiho Amano, Ryuichi Ohta, Chiaki Sano

**Affiliations:** 1 Community Care, Unnan City Hospital, Unnan, JPN; 2 Communiy Care, Unnan City Hospital, Unnan, Shimane, JPN; 3 Community Medicine Management, Faculty of Medicine, Shimane University, Izumo, JPN

**Keywords:** rural hospitals, hematologic malignancy, older individuals, differential diagnoses, intravascular lymphoma, natural killer t cell

## Abstract

Natural killer T cell intravascular lymphoma is a rare category of lymphoma among older individuals. The presentation of natural killer T cell lymphoma varies, causing diagnostic challenges for clinicians. Thus far, only a few studies have reported this condition in the context of musculoskeletal symptoms. We encountered a case of natural killer T cell intravascular lymphoma in a patient who presented with symptoms of sternoclavicular arthritis and femoral pain. The initial diagnosis was undifferentiated hematologic malignancy because undifferentiated hematologic malignant cells were seen on the bone marrow biopsy. Further examination showed that the patient had a high fever and abnormal cells in the blood. Flow cytometry findings revealed the abnormal cells as CD16 and CD56 positive, leading to the diagnosis of natural killer T cell intravascular lymphoma. This is the first report indicating the possibility of natural killer T cell intravascular lymphoma as one of the differential diagnoses of acute joint and muscular pains among older patients and the importance of assessing multiple organs, including musculoskeletal organs, to diagnose intravascular lymphoma.

## Introduction

Natural killer T (NKT) cell lymphoma is a rare category of lymphoma observed among older individuals. The presentation of NKT cell lymphoma varies, causing diagnostic challenges for clinicians [1]. T cells are initially divided into clusters of differentiation (CD) 4+ and CD8+ T cells; they kill infected or damaged cells, recognize antigens in the body, and stimulate B cells to generate antibodies against specific bacteria [2]. T-cells can also gain malignant status, and these malignant T-cells can invade various organs, causing systemic symptoms [3,4]. As lymphadenopathy is not a common feature of T cell lymphoma, diagnosis can be challenging [5,6]. The NKT cell, a subset of T cells, is versatile and kills not only various bacteria, but also infected and damaged cells [7]. When the NKT cell becomes malignant, the symptoms cannot be specific, making the diagnosis difficult.

Furthermore, NKT cell intravascular lymphoma may have a rare presentation among NKT cell lymphoma. As the identification of malignant NKT cells is difficult, NKT cell intravascular lymphoma eventually becomes leukemic [8,9]. So, it should be diagnosed by detecting malignant cells in the body. To diagnose the disease, searching the malignant cell in multiple organs is important. However, NKT cell intravascular lymphoma may not have lymphadenopathy and other organomegaly, which can make diagnosis even more complex [10].

To date, only a few studies regarding NKT cell intravascular lymphoma have reported this condition in the context of musculoskeletal symptoms [8,9]. We have encountered an older patient with fever and sternoclavicular joint pain, who was eventually diagnosed with NKT cell intravascular lymphoma. In the process of diagnosis, various tissues were tested including skin, blood, and bone marrow. We finally achieved a diagnosis after the disease had turned leukemic, and we detected malignant cells in the blood. In this report, we demonstrate the rare presentation of an NKT cell intravascular lymphoma. We also demonstrate the importance of follow-up using blood smear examination for diagnosis and decision-making for advanced care planning, in rural hospitals that lack medical resources. 

## Case presentation

An 87-year-old man presented with fever and left femoral pain at the emergency department of our hospital. A day before admission, he had pain in the lateral aspect of the left thigh. On the day of admission, he had a fever with chills, on being called for a home visit, his family doctor referred him to our hospital for further investigation and treatment, based on his condition. He had been taking prednisolone at a daily dose of 5 mg for the last three months to treat polymyalgia rheumatica and was receiving home oxygen therapy (2 L of O2) for chronic obstructive pulmonary disease.

**Figure 1:**
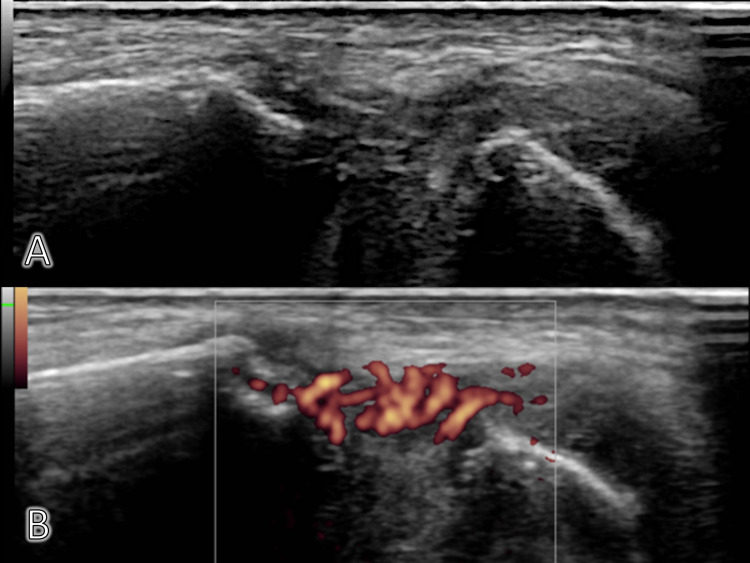
Ultrasound images of the left sternoclavicular joint The image shows fine flow in the joint space (A: Plain, B: With fine flow)

At the time of admission, his vital signs were as follows: body temperature, 38.4°C; blood pressure, 183/71 mmHg; heart rate, 82 beats/min; respiratory rate, 24 breaths/min; and SpO2, 95% on oxygen inhalation (2 L/min). He was alert and looked fatigued based on his general appearance. He had pain in the lateral aspect of his left thigh, bilateral sternoclavicular joints, and sternal body; purpura was seen at the site of pain in the sternoclavicular joints. Ultrasound examination showed soft tissue thickness with fine flow positive in bilateral sternoclavicular joints and the lateral surface of the left femur (Figure 1).

Completed blood count revealed leukocytosis (11,400 cells/μL, with differential counts showing 72% neutrophils, 6% lymphocytes, and 21% monocytes) and normocytic anemia (hemoglobin level: 6.8 g/dL, mean corpuscular volume: 92.7 fL). There was also an increased inflammatory reaction (C-reactive protein: 16.91 mg/dL, erythrocyte sedimentation rate: 110 mm). Lactate dehydrogenase (LDH) and ferritin levels were increased (LDH: 545 U/L, ferritin: 1,483.5 ng/mL) (Table 1).

**Table 1:** Initial laboratory data of the patient PT, prothrombin time; INR, international normalized ratio; APTT, activated partial thromboplastin time; UIBC, unsaturated iron-binding capacity; eGFR, estimated glomerular filtration rate; CK, creatine kinase; CRP, C-reactive protein; TSH, thyroid-stimulating hormone; T4, thyroxine; Ig, immunoglobulin; HBs, hepatitis B surface antigen; HBc, hepatitis B core antigen; HCV, hepatitis C virus; SARS-CoV-2, severe acute respiratory syndrome coronavirus 2; EBV VCA, Epstein-Barr virus capsid antigen; EBNA, Epstein-Barr virus nuclear antigen; HIV, human immunodeficiency virus; and CMV, cytomegalovirus

Maker	Level	Reference
White blood cells	11.4 × 10^3^	3.5–9.1 × 10^3^/μL
Neutrophils	72	44.0–72.0%
Lymphocytes	6	18.0–59.0%
Monocytes	21	0.0–12.0%
Eosinophils	0	0.0–10.0%
Basophils	0	0.0–3.0%
Red blood cells	2.2 × 10^6^	3.76–5.50 × 10^6^/μL
Reticulocytes (%)	5,304 (2.4)	/μL (%)
Hemoglobin	6.8	11.3–15.2 g/dL
Hematocrit	20.5	33.4–44.9%
Mean corpuscular volume	92.7	79.0–100.0 fL
Platelets	2.3 × 10^4^	13.0–36.9 × 10^4^/μL
PT-INR	1.09	
APTT	29.7	25–40 seconds
Fibrinogen	463.3	200–400 mg/dL
Fibrinogen degradation products	13.1	<5 μg/mL
Erythrocyte sedimentation rate	110	2–10 mm/hour
Total protein	7.7	6.5–8.3 g/dL
Albumin	3.6	3.8–5.3 g/dL
Total bilirubin	1.0	0.2–1.2 mg/dL
Direct bilirubin	0.2	0–0.4 mg/dL
Aspartate aminotransferase	19	8–38 IU/L
Alanine aminotransferase	14	4–43 IU/L
Alkaline phosphatase	239	106–322 U/L
γ-Glutamyl transpeptidase	56	<48 IU/L
Lactate dehydrogenase	545	121–245 U/L
Uric acid	4.5	3.0–6.9 mg/dL
Blood urea nitrogen	10.7	8–20 mg/dL
Creatinine	0.56	0.40–1.10 mg/dL
Serum Na	139	135–150 mEq/L
Serum K	3.4	3.5–5.3 mEq/L
Serum Cl	104	98–110 mEq/L
Serum Ca	9.7	3.5–10.2 mg/dL
Serum P	2.9	0.2–1.2 mg/dL
Serum Mg	2.1	1.8–2.3 mg/dL
Fe	22	54–181 μg/dL
UIBC	173	111–255 μg/dL
Ferritin	1483.5	14.4–303.7 ng/mL
CK	54	56–244 U/L
CRP	16.91	<0.30 mg/dL
Procalcitonin	0.18	0–0.05 ng/mL
TSH	1.61	0.35–4.94 μIU/mL
Free T4	1.4	0.70–1.48 ng/dL
Vitamin B12	459	180–914 pg/mL
Folic acid	7.1	>4.0 ng/mL
IgG	1087	870–1700 mg/dL
IgM	275	35–220 mg/dL
IgA	166	110–410 mg/dL
IgE	64	<173 mg/dL
HBs antigen	0.00	IU/mL
HBs antibody	0.79	mIU/mL
HBc antibody	0.08(-)	S/CO
HCV antibody	0.05	S/CO
Syphilis treponema antibody	0.04	S/CO
SARS-CoV-2 antigen	Negative	
EBV VCA IgG	8.0(+)	
EBV VCA IgM	0.4(-)	
EBV EBNA IgG	1.3(+)	
HIV antigen antibody	Negative	
CMV antigenemia	Negative	
Free light chain κ/λ	1.78	
Urine test		
Leukocyte	(-)	
Nitrite	(-)	
Protein	(+)	
Glucose	(-)	
Urobilinogen	(-)	
Bilirubin	(-)	
Ketone	(-)	
Blood	(+-)	
pH	7.0	
Specific gravity	1.017	
Fecal occult blood	(-)	

**Figure 2:**
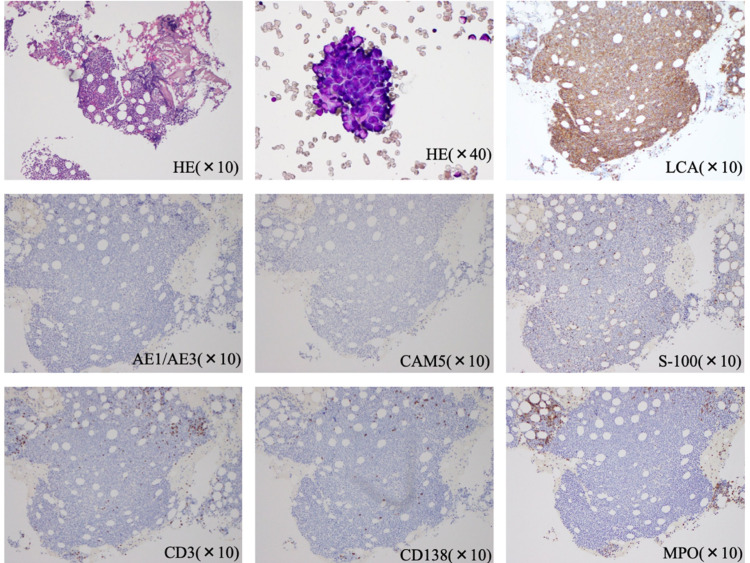
Pathology of the bone marrow Hematoxylin and eosin staining ×400 (HE) image showing leukocyte common antigen (LCA), Cytokeratin-multi-AE1/AE3 (AE1/AE3), Cytokeratin, CAM5 (CAM5), cluster of differentiation (CD), and myeloperoxidase (MPO)

There were no abnormal lymphocytes in peripheral blood. Tests for Epstein-Barr virus, cytomegalovirus, hepatitis B virus, and hepatitis C virus were negative. As we suspected lymphoma in the differential diagnosis, bone marrow aspiration and biopsy were performed. Findings on bone marrow examination showed diffuse infiltration of malignant cells with basophilic cytoplasm, anisonucleosis, and nucleoli with leukocyte common antigen positivity; they tested negative for creatine kinase, AE1/AE3, CAM5, S-100, CD3, CD20, CD138, and myeloperoxidase (MPO) (Figure 2).

**Figure 3:**
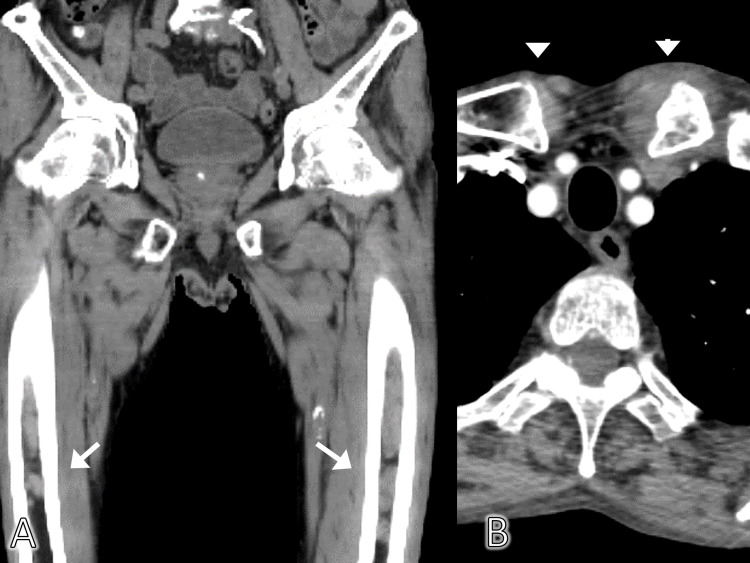
Contrast-enhanced computed tomography of the chest and computed tomography of the legs Computed tomography findings of the leg showed occupied lesions in the bone marrow of the bilateral femur and pelvis (A) with white arrows Contrast-enhanced computed tomography of the chest showing bilateral sternoclavicular joints (B) with white arrowheads

Random skin biopsy and upper and lower gastrointestinal tract endoscopy did not indicate any abnormality. Contrast-enhanced computed tomography of the neck, chest, abdomen, and pelvis showed bilateral sternoclavicular joint swelling and contrast enhancement of the proximal part of the left clavicle; bilateral pleural effusion, ascites, and occupied lesions in the bone marrow of bilateral femurs and the pelvis were also observed (Figure 3).

There was no hepatosplenomegaly or lymphadenopathy.

**Figure 4:**
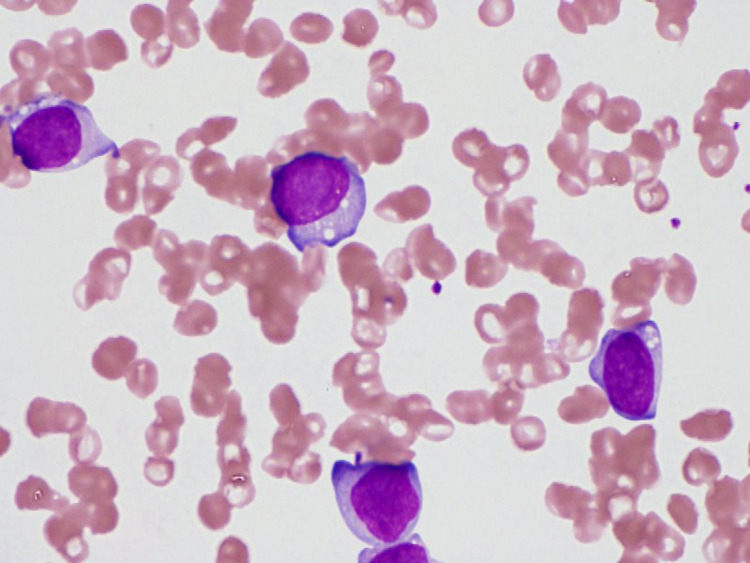
Peripheral blood smear image The image shows the appearance of blasts in the blood smear (May Grunwald Giemsa stain)

One week after admission, progression of anemia, the appearance of 10% blasts in the peripheral blood smear and an increase in the LDH levels were noted (Figure 4).

**Figure 5:**
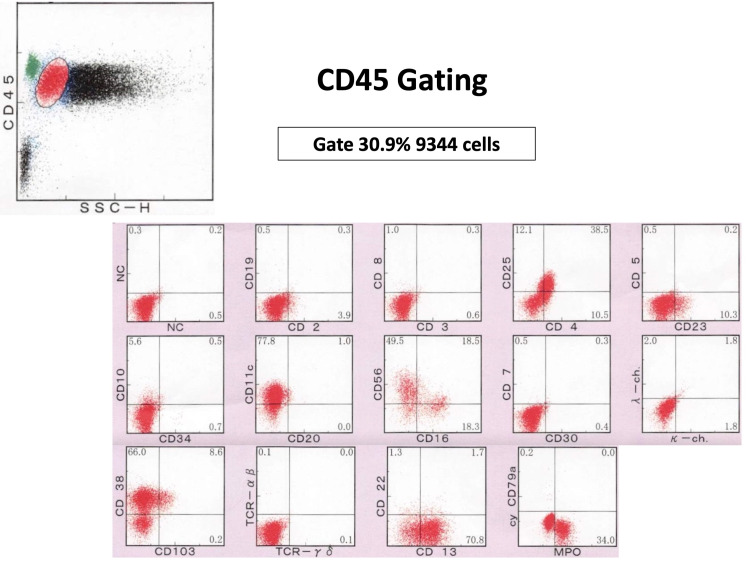
Flow cytometry images CD, cluster of differentiation; NC, negative control; TCR, T-cell receptor; MPO, myeloperoxidase; MPC-1, mitochondrial pyruvate carrier-1; SCC-H, side scatter height.

This was accompanied by a simultaneous deterioration in his condition; he became unconscious and hypoxemic, with a SpO2 of 92% on oxygen inhalation (5L/min), and fever with a body temperature of 40.1°C. As the clinical findings indicated leukemic changes from malignant lymphoma, flow cytometry of peripheral blood was performed. The findings showed a monoclonal increase in CD2-, CD3-, CD4±, CD5-, CD7-, CD8-, CD10-, CD19-, CD20-, cyCD3-, CD56+, CD16±, TCR-αβ-, TCR-γδ-, and MPO- cells, indicative of NKT cell lymphoma, which demonstrated CD 56 positivity and no T or B cell features (Figure 5).

He was suspected of having NKT cell intravascular lymphoma without blast cells at admission, along with lymphadenopathy and hepatosplenomegaly due to the bone marrow malignancy, and systemic bone and joint pains. Follow-up flow cytometry and peripheral blood studies showed an increase in blast cells suspected to be NKT cells; this led to a diagnosis of NKT cell intravascular lymphoma. The patient and his family were informed of the possibility of malignant disease and available chemotherapy; however, they preferred palliative care administered at home. He was offered symptomatic treatment with non-steroidal anti-inflammatory drugs and was discharged; he died at home a week later.

## Discussion

This case discusses the rare presentation of NKT cell intravascular lymphoma, and the importance of follow-up examination of blood smears for its diagnosis. Accurately diagnosing NKT cell intravascular lymphoma allows the medical team to have a frank discussion with the patient; this aids in understanding their goals and values, and in advanced planning of care.

NKT cell intravascular lymphoma causes various systemic symptoms; therefore, clinicians should suspect this disease based on multiple symptoms without specific origins. The presentation of NKT cell intravascular lymphoma varies [11]. The common symptoms are fever, fatigue, night sweat, and body weight loss; these are collectively termed B symptoms [11,12]. One of the rare presentations of NKT cell intravascular lymphoma is musculoskeletal pain [13]. Bone pain is typical, and involves long and large bones such as the femur and pelvic bones [14]; however, joint pain is rare. In this case, the patient has bilateral sternoclavicular joint arthritis. Although tissue investigation was not performed for the joints owing to the lack of fluid in that region, the joint pains appeared during disease progression without any evidence of gout or pseudogout. This case indicates that sternoclavicular joint pain can be one of the symptoms of NKT cell intravascular lymphoma.

NKT cell intravascular lymphoma can appear in various organs; hence, clinicians should continuously investigate various organs to detect malignant NKT cells. NKT cell intravascular lymphoma can be detected by random biopsy and bone marrow biopsy [1,10]. As the malignant cells may be overlooked in the first sample, clinicians should repeatedly investigate suspected organs. In this case, we detected malignant cells in the bone marrow, but could not diagnose the specific intravascular lymphoma due to the immaturity of the malignant cells. We could detect malignant cells in the blood based on blood smear findings and diagnose NKT cell lymphoma via follow-up flow cytometry when there was a sudden increase in the number of malignant cells. Intravascular lymphoma conditions can fluctuate and gradually deteriorate [10]. The transient exacerbation of symptomatic leukemic conditions may aid detection at diagnosis [12]. Cell counts determined by flow cytometry can also be beneficial for the diagnosis. In this case, we used the cell counts to diagnose NKT cell intravascular lymphoma. Rare cells can be difficult to detect on flow cytometry, owing to test characteristics of the categorization of a majority of cells. However, the transient increase in malignant cells in the blood can be analyzed through flow cytometry for diagnosis [10,12,15]. For the clinical diagnosis of NKT cell intravascular lymphoma, clinicians should focus on the frequency of blasts in the blood and cell counts on CD marker analysis during flow cytometry.

This case emphasizes the need for suspecting the diagnosis in advance based on certain symptoms, planning for a specific diagnosis, and the importance of specifying the diagnosis for advanced care planning. Achieving a diagnosis can be critical for advanced care planning, and can drive clinician-guided decision-making for patients and their families [16]. Both the diagnosis and treatment of NKT cell intravascular lymphoma can be challenging, especially among the older population, owing to their longevity and daily activities [12]. The mortality rate of the condition within one year is known to be 58.6%; the presence of B symptoms, or other symptoms except for cutaneous manifestations, confer higher mortality despite receipt of CHOP (cyclophosphamide, doxorubicin, vincristine, and prednisone) and DHAP (dexamethasone, cytarabine, and cisplatin) chemotherapy [12]. Although there are no studies regarding mortality among older people with NKT cell intravascular lymphoma, findings from previous research indicate that the mortality may be high [9]. Nevertheless, obtaining a clear diagnosis is important for effective decision making among patients and guiding the selection of palliative care [17]; in this case, the patient and his family chose home care owing to their limitations, and to allow him to spend more time together with his family during the limited time available. Based on these considerations, it is important that the diagnostic process of NKT cell intravascular lymphoma is logically organized in rural hospitals. This case shows the challenges and the possibilities for diagnosing NKT cell intravascular lymphoma. In urban and advanced hospitals, the use of positron emission tomography may allow detection of NKT cell intravascular lymphoma foci earlier. However, limited medical resources and fewer medical tests hinder chances of flow cytometry-based assessment of the bone marrow on initial presentation. As survival among older individuals with NKT cell intravascular lymphoma can be limited, it is important to be aware of its possibility; this may aid the detection of blasts in the blood, leading to an appropriate diagnosis and advanced planning of care.

## Conclusions

This case demonstrates a rare presentation of NKT cell intravascular lymphoma, and the importance of follow-up examination using blood smears for diagnosis and decision-making for advanced care planning. Meticulous monitoring of the disease and planning for the diagnosis is essential for the diagnosis of NKT cell intravascular lymphoma. An increasing number of older people residing in rural areas do not intend to be transferred to urban hospitals. Therefore, it is essential that even clinicians in rural community hospitals prepare for diagnosing hematological malignancies based on both, cell types and phenotypes.
